# Perineural Epinephrine for Brachial Plexus Block Increases the Incidence of Hypotension during Dexmedetomidine Infusion: A Single-Center, Randomized, Controlled Trial

**DOI:** 10.3390/jcm10122579

**Published:** 2021-06-11

**Authors:** Chahyun Oh, Boohwi Hong, Yumin Jo, Seungbin Jeon, Sooyong Park, Woosuk Chung, Youngkwon Ko, Sun Yeul Lee, Chaeseong Lim

**Affiliations:** 1 Department of Anesthesiology and Pain Medicine, Chungnam National University Hospital, Daejeon 35015, Korea; ohchahyun@gmail.com (C.O.); koho0127@gmail.com (B.H.); lemonny87@naver.com (Y.J.); dasom4924@naver.com (S.J.); sycom7@gmail.com (S.P.); woosuk119@gmail.com (W.C.); annn8432@gmail.com (Y.K.); 2 Department of Anesthesiology and Pain Medicine, College of Medicine, Chungnam National University, Daejeon 35015, Korea

**Keywords:** epinephrine, dexmedetomidine, regional anesthesia, brachial plexus block, sedation, hypotension

## Abstract

Background: Sedation using dexmedetomidine is frequently associated with hypotension. In contrast, epinephrine, a commonly used adjunctive agent in regional anesthesia, is a potent vasopressor. We hypothesized that perineural epinephrine used in brachial plexus blockade may reduce hypotension during dexmedetomidine infusion. Methods: Patients scheduled for upper extremity surgery were randomly allocated into a control and an epinephrine group. All patients received brachial plexus blockade, consisting of 25 mL of a 1:1 mixture of 1% lidocaine and 0.75% ropivacaine, with patients in the epinephrine group also receiving 125 μg epinephrine. Intraoperative sedation was induced using dexmedetomidine at a loading dose of 1 µg/kg and maintenance dose of 0.4 µg/kg/hr. The primary outcome was the incidence of intraoperative hypotension or hypotension in the post-anesthesia care unit (PACU). Results: One hundred and thirty patients were included (65 per group). The incidence of hypotension was significantly higher in the epinephrine than in the control group (80.6% vs. 56.9%, *p* = 0.009). The duration of hypotension and the maximal change in blood pressure were also greater in the epinephrine group. Conclusions: Perineural epinephrine for brachial plexus blockade does not reduce hypotension due to dexmedetomidine infusion and may actually augment the occurrence of hypotensive events.

## 1. Introduction

Dexmedetomidine, an alpha-2 adrenergic agonist with a unique sedative effect, known as arousable sedation, is widely used in regional anesthesia due to its favorable clinical profile. Unlike other sedatives, it enables cooperative sedation while preserving respiratory drive [1,2]. Intraoperative dexmedetomidine also has its own analgesic and opioid-sparing effects [3,4,5]. However, dexmedetomidine infusion can also induce undesirable hemodynamic effects, such as an initial transient increase in blood pressure due to peripheral vasoconstriction, followed by a continuous decrease in blood pressure, predominantly through vasodilatory effects acting via central alpha-2A receptors [2].

Epinephrine is another drug often used during regional anesthesia due to its vasoconstrictive effect, which may delay the systemic uptake of local anesthetic and prolong the blockade when used as an adjunctive agent for peripheral nerve blockade [6]. Its chronotropic properties also serve for surveillance of accidental intravascular injection during the blockade [7].

Because dexmedetomidine and epinephrine have opposing hemodynamic properties, the use of epinephrine as an adjunctive agent during peripheral nerve blockade may prevent the decrease in blood pressure caused by dexmedetomidine infusion. To date, however, no studies have assessed the hemodynamic interactions of these two agents when used together as adjuvants during regional anesthesia. The present study therefore evaluated the hemodynamic effects of perineural epinephrine during dexmedetomidine infusions in patients undergoing upper extremity surgery under a brachial plexus block.

## 2. Materials and Methods

This randomized, controlled, double-blinded trial was conducted at the Chungnam National University Hospital, Korea, from May 2020 to January 2021. The study was approved by the Chungnam National University Hospital’s Institutional Review Board (CNUH 2020-03-100) and was registered prior to patient enrollment at cris.nih.go.kr (KCT0004961). Study data were collected and managed using the REDCap (Research Electronic Data Capture) software hosted at Chungnam National University Hospital. The REDCap is a secure, web-based platform designed to support capturing of data for research studies [8]. This study also adhered to the applicable CONSORT (Consolidated Standards of Reporting Trials) guidelines [9].

### 2.1. Study Participants

Patients were evaluated for study eligibility, and written informed consent was obtained from all enrolled participants before surgery. Patients aged 20–65 years with American Society of Anesthesiologists (ASA) physical status I or II scheduled for orthopedic upper extremity surgery under regional anesthesia and sedation were recruited. Exclusion criteria included hypersensitivity to local anesthetic, epinephrine or dexmedetomidine; coagulopathy or local infection at the block site; hemodynamic instability, such as uncontrolled hypertension or tachy- or bradycardia; and patient refusal. Shoulder surgery was also excluded since it is commonly performed under general anesthesia with sitting position in our institution. Uncontrolled hypertension was defined as systolic blood pressure >160 mmHg, determined as the average of two or three available preoperative measurements.

### 2.2. Randomization and Minimization of Bias

Block randomization with sizes of 2 and 4 was performed with a random sequence generator (www.randomization.com, accessed on 11 May 2020). To conceal the allocation, the sequence was uploaded to REDCap (version 6.11.5, redcap.cnuh.co.kr), allowing access only to the researcher preparing the assigned drug for brachial plexus block. All other individuals who participated in the surgery, including the attending anesthesiologist, the patient, and the outcome assessor, were blinded to group assignment.

Patients were randomly assigned 1:1 to a control group or an epinephrine group. Patients in the control group received 24 mL of a 1:1 mixture of 1% lidocaine and 0.75% ropivacaine, plus 1 mL of 0.9% saline, whereas patients in the epinephrine group received the same mixture of lidocaine and ropivacaine plus 125 μg of epinephrine diluted in 1 mL of 0.9% saline. These mixtures were prepared in visually identical 30 mL syringes immediately before the patients entered the operating theater, and, depending on the assignment, handed to the attending anesthesiologist performing the blockade.

### 2.3. Anesthetic Procedures

Standard ASA monitoring was applied before performing the block and maintained throughout the entire procedure. All blocks were performed without any supplemental sedative under ultrasound guidance using an in-plane technique with a high-resolution ultrasound system (X-Porte, FUJIFILM Sono Site, Inc, Bothell, WA, USA), a high-frequency linear probe (HFL50xp: 15–6 MHz, X-Porte) and a nerve stimulator (0.1 ms, 0.5 mA, 2 Hz, sentinel mode, MultiStim SENSOR, PAJUNK, Germany). The type of blockade (supraclavicular, costoclavicular, axillary) was determined by the attending anesthesiologist. Interscalene block was not included in the protocol since the blockade is not commonly used for upper extremity surgery other than shoulder surgery in our institution to avoid the risk of hemidiaphragmatic paralysis and ulnar sparing. When the surgical field extended to the medial side of the upper arm, an additional intercostobrachial nerve blockade was performed using 5 to 10 mL of 0.25% ropivacaine. Dexmedetomidine infusion was initiated only after confirming the onset of hypoesthesia in terminal nerve dermatomes (i.e., radial, median, ulnar, musculocutaneous) related to the operating field. Dexmedetomidine was infused at a loading dose of 1 µg/kg over 10 min and maintained at a dose of 0.4 µg/kg/h. Supplemental oxygen during the sedation was administered at a rate of 5 L/min via a simple facial mask. Dexmedetomidine was discontinued at the beginning of skin suture. In case of significant hemodynamic compromise such as severe bradycardia or hypotension, the infusion rate of dexmedetomidine was adjusted or small doses of vasopressors (i.e., aliquots of 5 mg intravenous ephedrine, 50 µg phenylephrine) or 0.5 mg atropine were administered. Although not prespecified, small doses of supplementary sedatives (2–3 mg midazolam or 20–30 mg propofol) or opioid (20–30 µg fentanyl) were allowed. The decision to intervene was made by the attending anesthesiologist.

### 2.4. Outcome Measures

Except for heart rate, which was monitored continuously by pulse oximetry, all other hemodynamic variables (systolic, mean, and diastolic blood pressure) were measured at 5 min intervals with a non-invasive blood pressure cuff applied to the upper extremity not undergoing surgery. The primary outcome was the incidence of hypotension during and after surgery (i.e., during stay in the post-anesthesia care unit [PACU]). Hypotension was defined as mean blood pressure <60 mmHg; a >25% reduction in mean blood pressure; systolic blood pressure <90 mmHg; or a >25% reduction in systolic blood pressure. The baseline hemodynamic variables were set by averaging two to three measurements obtained in the ward before arrival in the operating room.

Secondary outcomes included the occurrence of bradycardia, defined as <75% of baseline and <50 beats per minute; maximum reduction of systolic, mean, and diastolic blood pressure; drug administration due to hemodynamic compromise; length of stay in the PACU; and first request for rescue analgesics.

### 2.5. Statistical Analysis

The sample size of this study was determined based on the results of a retrospective review of vital records of patients at our institution who underwent brachial plexus blockade and sedation using dexmedetomidine in 2019. The incidence of hypotension in these patients was about 30%. Based on the assumption that the incidence of hypotension in the epinephrine group would be 10%, each group should have 59 subjects to have a power of 80% and a risk of 5% for type I error. To account for potential dropouts and losses of data, this study aimed to recruit 130 subjects (65 per group).

Results were analyzed on both an intention-to-treat (ITT) and a per-protocol (PP) basis using R software version 4.0.2 (R Project for Statistical Computing, Vienna, Austria). Continuous variables were reported as the mean ± standard deviation (SD) and analyzed by independent sample t-tests or as median (interquartile range (IQR)) and analyzed by Mann-Whitney U tests, depending on the results of Shapiro-Wilk tests. Categorical variables were reported as number (%) and analyzed using χ^2^ or Fisher’s exact (expected count < 5). A two-tailed *p*-value < 0.05 was considered statistically significant. Intergroup comparisons of time series data of hemodynamic variables were performed using a generalized additive mixed model with random intercept. This model included an interaction (smooth) term between time (12 time points, including the initial measurement in the operating room and 11 subsequent time points in 5 min intervals) and group as a fixed effect, and individual (smooth term) as a random effect.

## 3. Results

**Figure 1:**
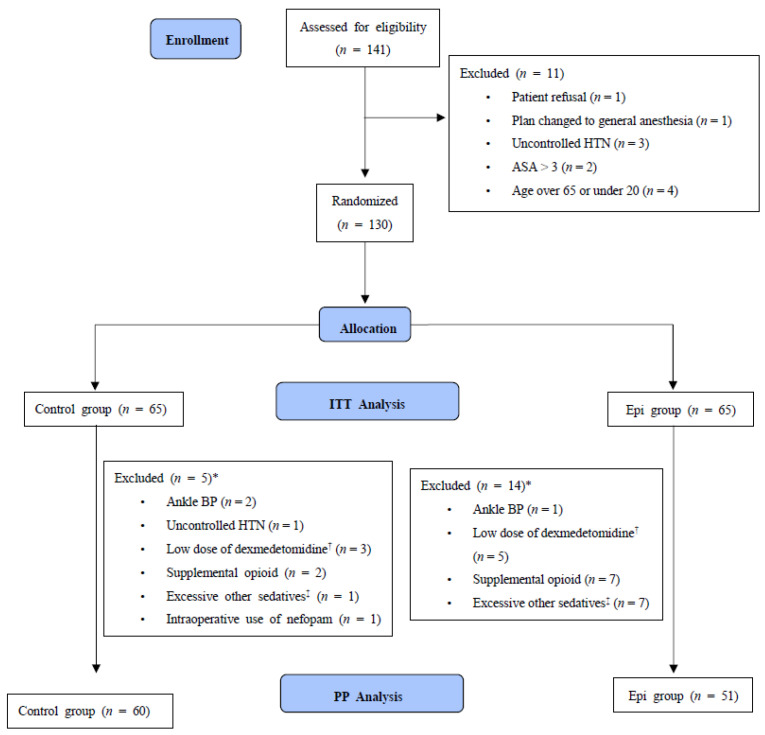
Consolidated Standards of Reporting Trials (CONSORT) flow chart. * The count is the sum of the cases including duplicated reasons for exclusion. ^†^ Less than 90% of loading dose; 0.9 mcg/predicted body weight (kg). ^‡^ Propofol over 30 mg or midazolam over 3 mg. HTN: hypertension, ASA: American Society of Anesthesiologist physical status, ITT: intention-to-treat, BP: blood pressure, PP: per-protocol.

Study eligibility was assessed in 141 patients; of these, 11 patients were excluded, and the remaining 130 patients were randomized into two groups of 65 patients each. All 130 patients were included in the ITT analysis, whereas 111 (60 in the control group and 51 in the epinephrine group) were included in the PP analysis. PP analysis was performed after excluding patients with significant deviations from the predefined study protocol. Significant deviations were determined at the analysis stage based on the following criteria: uncontrolled hypertension (initial systolic blood pressure in the operating room >190 mmHg); low dosage of dexmedetomidine (<90% of loading dose; or 0.9 µg/kg predicted body weight); supplemental opioid use; excessive use of another sedative (>30 mg propofol or >3 mg midazolam) due to patient discomfort or unexpected or uncooperative movements during the procedure; inability to use the contralateral arm for blood pressure measurements; or the use of drugs (e.g., nefopam) that could affect hemodynamic parameters (Figure 1). The demographic and clinical characteristics of the patients included in the ITT population are shown in Table 1.

The results of the ITT and PP analyses of the primary outcome are summarized in Table 2. The incidence of hypotension (intra- and postoperative) was significantly higher in the epinephrine group on ITT analysis (*p* = 0.009). However, the difference in the PP population did not reach statistical significance (*p* = 0.050). Secondary outcomes are summarized in Table 3. The duration of hypotension and the maximal changes in hemodynamic variables (systolic, mean, diastolic blood pressure and heart rate) relative to baseline differed significantly in the two groups. In contrast, the incidences of bradycardia and drug use due to hemodynamic compromise, as well as the length of stay in the PACU, did not differ significantly between these two groups. There was no repeated administration of vasopressor or atropine.

**Figure 2:**
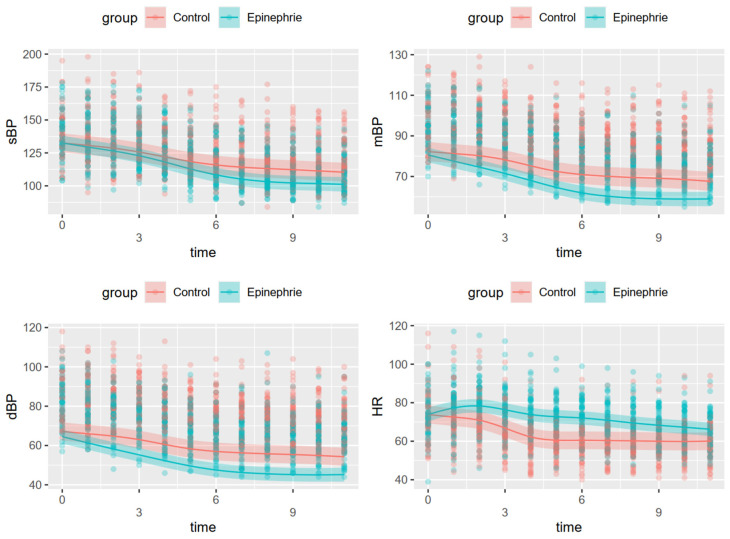
Hemodynamic changes over time. Time (0) indicates initial measurement in the operating room. Time (1) indicates the initiation of dexmedetomidine infusion, with times 1–11 being at intervals of 5 min each. sBP: systolic blood pressure, mBP: mean blood pressure, dBP: diastolic blood pressure, HR: heart rate.

The results of all four generalized additive mixed models for each hemodynamic variable are summarized in Table 4 and Figure 2. Changes in the four variables differed significantly between the two groups. The epinephrine group showed a modest increase in heart rate from the initiation of blockade to 15 min after the initiation of dexmedetomidine infusion. Unexpectedly, the decrease in blood pressure during dexmedetomidine infusion was greater in the epinephrine than in the control group.

## 4. Discussion

The results of the present suggest that the use of epinephrine as an adjunctive agent for brachial plexus blockade does not prevent hypotension induced by dexmedetomidine infusion. In fact, contrary to our hypothesis, the use of epinephrine actually increased the occurrence of hypotensive events. Because negative outcomes are associated with perioperative hypotension [10,11,12,13], our results suggest that anesthesiologists must be aware of the increased likelihood of hypotension when using both perineural epinephrine and dexmedetomidine.

Our primary hypothesis was based on the hemodynamic effects of epinephrine. As a widely used vasopressor, we assumed that perineural epinephrine could compensate for the hemodynamic changes caused by dexmedetomidine. Few previous studies, however, have assessed the hemodynamic effects of perineural injection of epinephrine during brachial plexus block. One such study reported that both heart rate and blood pressure increased when epinephrine was used as an adjuvant during axillary block [14]. However, these changes in hemodynamics were monitored only for 10 min. Most importantly, the ability of perineural epinephrine to counter the hemodynamic changes induced by sedative agents has not yet been evaluated. To our knowledge, the present results are the first to reveal a synergetic hypotensive effect between perineural epinephrine and dexmedetomidine for brachial plexus block.

Although our results were unexpected, epinephrine has been reported to induce hypotension in both humans and rats [15,16,17]. This seemingly paradoxical hypotension induced by epinephrine, a potent vasopressor, may be explained by vasodilation mediated by beta-2 adrenergic receptor activation [18,19,20]. Because skeletal muscle exerts beta-dominant adrenergic effects [21], muscles surrounding the injection site for the blockade, such as the omohyoid and scalene muscles, may contribute to this phenomenon. In addition, dexmedetomidine may inhibit the alpha-adrenergic effect of epinephrine, as well as contributing to its beta-dominant effect [22]. These findings are in agreement with our present results, including the steeper reductions in mean and diastolic blood pressure and the modest increase in heart rate observed in the epinephrine group.

The association of negative patient outcomes with perioperative hypotension [10,11,12,13] suggests the need for minimum blood pressure targets, such as a mean blood pressure of 60 to 65 mmHg or a systolic blood pressure of 80 to 90 mmHg. Even if these values are based on robust evidence, an absolute target may not be sufficient to optimize individual blood pressure management [23,24]. The present study set comprehensive criteria for hypotension using relative and absolute values. Despite mean blood pressure not being lower than 60 mmHg during most of the hypotensive events occurring in the current study, clinical consideration is required [25].

This study had several limitations. First, the infusion rate of dexmedetomidine varied among patients. Because the loading dose accounted for most of the total dose, there was no significant between-group difference in the total consumption of dexmedetomidine, measured as µg/kg. Second, other sedative and analgesic agents were administered to these patients to optimize patient comfort. Nevertheless, PP analysis found that the incidence of intraoperative hypotension was about twice as high in the epinephrine as in the control group, suggesting that the use of additional sedatives or analgesics did not alter our results. Third, hypotension was defined using both relative and absolute values, as previous studies suggested that an absolute target may not be sufficient to optimize individual blood pressure management. Thus, the incidence of hypotension may be dependent on the criteria used for its definition. Fourth, the use of vasopressor or atropine was not standardized due to the lack of a pre-specified threshold for the use of these drugs. However, since the incidence of the drug use was only 4 (6.2%) and 1 (1.5%) in the control and the epinephrine group, the effects of such non-standardized administration may not be significant.

## 5. Conclusions

In conclusion, adjunctive use of epinephrine for brachial plexus blockade can increase the incidence of hypotension during dexmedetomidine infusion. When concurrent use is planned, a different dosing of dexmedetomidine, such as omitting the initial loading dose or slow titration, should be considered. Further research is warranted regarding the potential interactions between dexmedetomidine and epinephrine.

## Figures and Tables

**Table 1:** Demographics and clinical characteristics of the study patients.

	Control	Epinephrine
	(*n* = 65)	(*n* = 65)
Age (yr), median (IQR)	46.0 (31.0–57.0)	50.0 (38.0–57.0)
Sex (M/F)	26/39	25/40
Height (cm), median (IQR)	164.0 (160.0–173.0)	167.0 (158.0–174.4)
Weight (kg), mean ± SD	68.2 ± 11.6	69.4 ± 13.9
BMI (kg/m^2^), mean ± SD	24.7 ± 2.8	24.9 ± 3.5
ASA (1/2)	14/51	4/61
Diabetes mellitus	5 (7.7%)	4 (6.2%)
Hypertension	13 (20.0%)	13 (20.0%)
Cardiovascular disease	1 (1.5%)	0 (0.0%)
Anesthesia time (min), median (IQR)	85.0 (64.0–112.0)	75.0 (58.0–104.0)
Type of procedure		
- Elbow	10 (15.4%)	8 (12.3%)
- Forearm	26 (40.0%)	24 (36.9%)
- Hand	19 (29.2%)	25 (38.5%)
- Wrist	10 (15.4%)	8 (12.3%)
Type of blockade		
- Axillary	1 (1.5%)	2 (3.1%)
- Costoclavicular	5 (7.7%)	8 (12.3%)
- Supraclavicular	59 (90.8%)	55 (84.6%)
Dexmedetomidine (µg/kg), median (IQR)	1.3 (1.1–1.5)	1.2 (1.1–1.4)
Baseline sBP (mmHg), mean ± SD	126.5 ± 12.8	127.8 ± 11.4
Baseline mBP (mmHg), mean ± SD	92.7 ± 9.9	93.9 ± 8.1
Baseline dBP (mmHg), mean ± SD	75.7 ± 9.7	76.9 ± 8.1
Baseline heart rate (beats/min), mean ± SD	73.9 ± 9.9	74.1 ± 8.7

BMI: body mass index, ASA: American Society of Anesthesiologist physical status, sBP: systolic blood pressure, mBP: mean blood pressure, dBP: diastolic blood pressure, HR: heart rate, LOS: length of stay, PACU: post-anesthesia care unit.

**Table 2:** Incidence of hypotension in the study patients.

	ITT		*p*	PP		*p*
	Control	Epinephrine		Control	Epinephrine	
	(*n* = 65)	(*n* = 65)		(*n* = 60)	(*n* = 51)	
Hypotension	33 (56.9%)	50 (80.6%)	0.009	31 (57.4%)	38 (77.6%)	0.05
Intraoperative	15 (23.1%)	35 (53.8%)	0.001	14 (23.3%)	28 (54.9%)	0.001
Postoperative	30 (52.6%)	42 (71.2%)	0.062	29 (54.7%)	32 (69.6%)	0.191

ITT: intention-to-treat, PP: per-protocol.

**Table 3:** Secondary outcomes in the study population.

	Control	Epinephrine	*p*
	(*n* = 65)	(*n* = 65)	
Hypotension duration (min), median (IQR)	5.0 (0.0–35.0)	20.0 (5.0–55.0)	0.003
Bradycardia, *n* (%)	11 (18.6%)	7 (11.9%)	0.442
- Intraoperative	10 (15.4%)	7 (11.9%)	0.603
- Postoperative	7 (12.3%)	3 (5.1%)	0.294
Maximum change, mean ± SD			
- sBP, mmHg	26.1 ± 12.5	30.6 ± 12.3	0.039
- mBP, mmHg	23.4 ± 10.3	27.0 ± 10.5	0.047
- dBP, mmHg	16.8 ± 10.2	20.8 ± 10.1	0.027
Heart rate, beats/min	19.3 ± 7.0	15.8 ± 9.0	0.014
Drug use *, *n* (%)	4 (6.2)	1 (1.5)	0.362
LOS in PACU (hr), median (IQR)	36.0 (34.0–43.0)	36.0 (33.0–41.0)	0.754
Time to first rescue analgesic ** (min), mean ± SD	714.8 ± 195.0	654.3 ± 210.0	0.292

* The number of cases in which the drug (aliquots of 5 mg intravenous ephedrine or 50 µg phenylephrine or 0.5 mg atropine) is administered due to hemodynamic compromise. There was no repeated administration of the drug. ** Patients not using patient-controlled analgesia (*n* = 47 in the control group; *n* = 51 in the epinephrine group). sBP: systolic blood pressure, mBP: mean blood pressure, dBP: diastolic blood pressure, HR: heart rate, LOS: length of stay, PACU: post-anesthesia care unit.

**Table 4:** Summary of the four generalized additive mixed models.

Dependent Variable	Systolic Blood Pressure			Mean Blood Pressure			Diastolic Blood Pressure			Heart Rate		
Adjusted R^2^	0.752			0.776			0.763			0.775		
**Parametric Coefficient**	**Estimate**	**SE**	***p***	**Estimate**	**SE**	***p***	**Estimate**	**SE**	***p***	**Estimate**	**SE**	***p***
Intercept	127.294	1.697	<0.001	88.883	1.116	<0.001	77.401	1.087	<0.001	63.327	1.113	<0.001
Group (epinephrine)	–6.019	2.400	0.012	–7.596	1.578	<0.001	–8.188	1.538	<0.001	8.286	1.575	<0.001
**Smooth Terms**	**EDF**	**F**	***p***	**EDF**	**F**	***p***	**EDF**	**F**	***p***	**EDF**	**F**	***p***
Time by Control G	3.991	97.87	<0.001	4.797	88.77	<0.001	4.321	73.97	<0.001	6.411	82.45	<0.001
Time by Epinephrine G	4.523	191.01	<0.001	4.435	212.17	<0.001	4.087	176.08	<0.001	6.308	44.71	<0.001
Individual	122.756	23.30	<0.001	122.903	23.82	<0.001	122.749	23.28	<0.001	123.259	27.66	<0.001

SE: standard error, EDF: estimated degrees of freedom, G: group.

## Data Availability

The data presented in this study are available on request from the corresponding author.
